# Prophage-Mediated Disruption of Genetic Competence in Staphylococcus pseudintermedius

**DOI:** 10.1128/mSystems.00684-19

**Published:** 2020-02-18

**Authors:** Michael R. Brooks, Lyan Padilla-Vélez, Tarannum A. Khan, Azaan A. Qureshi, Jason B. Pieper, Carol W. Maddox, Md Tauqeer Alam

**Affiliations:** a Department of Pathobiology, College of Veterinary Medicine, University of Illinois at Urbana-Champaign, Urbana, Illinois, USA; b Department of Veterinary Clinical Medicine, College of Veterinary Medicine, University of Illinois at Urbana-Champaign, Urbana, Illinois, USA; UMR1136 INRA Université de Lorraine

**Keywords:** *Staphylococcus*, antibiotic resistance, bacteriophage evolution, drug resistance evolution, genetic competence, genomics

## Abstract

Methicillin-resistant Staphylococcus pseudintermedius (MRSP) is a major cause of soft tissue infections in dogs and occasionally infects humans. Hypervirulent multidrug-resistant (MDR) MRSP clones have emerged globally. The sequence types ST71 and ST68, the major epidemic clones of Europe and North America, respectively, have spread to other regions. The genetic factors underlying the success of these clones have not been investigated thoroughly. Here, we performed a comprehensive genomic analysis of 371 S. pseudintermedius isolates to dissect the differences between major clonal lineages. We show that the prevalence of genes associated with antibiotic resistance, virulence, prophages, restriction-modification (RM), and CRISPR/Cas systems differs significantly among MRSP clones. The isolates with GyrA+GrlA mutations, conferring fluoroquinolone resistance, carry more of these genes than those without GyrA+GrlA mutations. ST71 and ST68 clones carry lineage-specific prophages with genes that are likely associated with their increased fitness and virulence. We have discovered that a prophage, SpST71A, is inserted within the *comGA* gene of the late competence operon *comG* in the ST71 lineage. A functional *comG* is essential for natural genetic competence, which is one of the major modes of horizontal gene transfer (HGT) in bacteria. The RM and CRISPR/Cas systems, both major genetic barriers to HGT, are also lineage specific. Clones harboring CRISPR/Cas or a prophage-disrupted *comG* exhibited less genetic diversity and lower rates of recombination than clones lacking these systems. After Listeria monocytogenes, this is the second example of prophage-mediated competence disruption reported in any bacteria. These findings are important for understanding the evolution and clonal expansion of MDR MRSP clones.

**IMPORTANCE** Staphylococcus pseudintermedius is a bacterium responsible for clinically important infections in dogs and can infect humans. In this study, we performed genomic analysis of 371 S. pseudintermedius isolates to understand the evolution of antibiotic resistance and virulence in this organism. The analysis covered significant reported clones, including ST71 and ST68, the major epidemic clones of Europe and North America, respectively. We show that the prevalence of genes associated with antibiotic resistance, virulence, prophages, and horizontal gene transfer differs among clones. ST71 and ST68 carry prophages with novel virulence and antibiotic resistance genes. Importantly, site-specific integration of a prophage, SpST71A, has led to the disruption of the genetic competence operon *comG* in ST71 clone. A functional *comG* is essential for the natural uptake of foreign DNA and thus plays an important role in the evolution of bacteria. This study provides insight into the emergence and evolution of antibiotic resistance and virulence in S. pseudintermedius, which may help in efforts to combat this pathogen.

## Abstract (precis)

Staphylococcus pseudintermedius is a bacterium responsible for clinically important infections in dogs and can infect humans. In this study, we performed genomic analysis of 371 S. pseudintermedius isolates to understand the evolution of antibiotic resistance and virulence in this organism. The analysis covered significant reported clones, including ST71 and ST68, the major epidemic clones of Europe and North America, respectively. We show that the prevalence of genes associated with antibiotic resistance, virulence, prophages, and horizontal gene transfer differs among clones. ST71 and ST68 carry prophages with novel virulence and antibiotic resistance genes. Importantly, site-specific integration of a prophage, SpST71A, has led to the disruption of the genetic competence operon *comG* in ST71 clone. A functional *comG* is essential for the natural uptake of foreign DNA and thus plays an important role in the evolution of bacteria. This study provides insight into the emergence and evolution of antibiotic resistance and virulence in S. pseudintermedius, which may help in efforts to combat this pathogen.

## INTRODUCTION

Staphylococcus pseudintermedius is a leading cause of skin, postoperative, ear, and urinary tract infections in dogs (1). Although S. pseudintermedius does not typically colonize humans, there have been sporadic cases of S. pseudintermedius transmission from dogs to humans, confirming its ability to colonize and cause infections in humans (2, 3). Approximately 5% of dog owners are estimated to carry S. pseudintermedius on their skin or nose, which upon infection, can cause symptoms similar to those in dogs (4, 5). Since its first documented appearance in the late 1990s, the prevalence of methicillin-resistant S. pseudintermedius (MRSP) cases in dogs has been increasing at an alarming rate (6–9). MRSP strains resistant to multiple classes of antibiotics (multidrug resistant [MDR)] have emerged globally, including in the United States, Australia, New Zealand, Canada, and countries in Europe and Asia (8, 10–12). These MDR MRSP strains are often found to carry fluoroquinolone resistance (FQR)-conferring GyrA Ser84Leu and GrlA Ser80Ile mutations, along with other acquired genes associated with aminoglycoside, macrolide, lincosamide, tetracycline, and trimethoprim-sulfamethoxazole resistance. MDR MRSP infections are difficult to treat because they do not respond to commonly available antibiotics in veterinary medicine (13). The rapid evolution and global spread of the MDR MRSP clones ST71, ST68, and ST45 are making the situation worse (7, 10, 14).

Studies utilizing multilocus sequence typing (MLST) and whole-genome sequencing (WGS) have demonstrated that the S. pseudintermedius population is genetically diverse, with more than 1,400 sequence types (STs) reported to date (7, 10, 15). The isolates belonging to different STs differ significantly from each other in their geographical prevalence, antibiotic resistance pattern, virulence gene prevalence, and the types of staphylococcal cassette chromosome *mec* (SCC*mec*) elements they carry (14, 16–18). For example, S. pseudintermedius ST71 *SCCmec* II-III is the most predominant MDR MRSP clone in European countries, with ST258, ST261, and ST496 being the other frequently reported clones (7, 17). ST68 SCC*mec* V is the most common MDR MRSP clone in the United States; however, other frequently reported clones include ST64, ST71, ST84, ST150, ST155, ST181, and ST1049 (7, 10, 18–20). Recent studies confirm the widespread presence of the European clone ST71 in the United States, Australia, New Zealand, Asia, and South America (8, 10, 12, 20). Similarly, the North American clone ST68 has been reported in Europe and Asia (10). ST45 is another highly successful MDR MRSP clone that has spread to Asia, North America, Europe, and Australia (10, 21, 22). From previous studies, it is clear that ST71, ST68, and ST45 are the most successful and rampant S. pseudintermedius clones. However, the underlying genetic factors contributing to their evolutionary success and global dissemination have not been fully investigated. We hypothesize that these epidemic clones may have acquired unique mobile genetic elements (MGEs), genes associated with virulence, antibiotic resistance, and other advantageous changes. The clonal expansion and evolution of many bacterial pathogens, including Staphylococcus aureus, Streptococcus pyogenes, and Streptococcus agalactiae have been driven by the acquisition of antibiotic resistance-conferring mutations and MGEs (23–25). Therefore, a comprehensive genomic analysis of all major S. pseudintermedius clones is needed to better understand the emergence and evolution of multidrug resistance and virulence in this pathogen. Genomic studies thus far have analyzed only a limited number of country-specific sequence types and have focused on selected antibiotic resistance and virulence genes (8, 11, 12, 14, 17, 20, 21, 26, 27).

Here, we report a comprehensive analysis of 371 S. pseudintermedius genomes representing all major MDR MRSP clones. We have identified several lineage-specific genetic features in S. pseudintermedius, including prophages and genes associated with antibiotic resistance, virulence, and horizontal gene transfer (HGT). For the first time, we have discovered that the European MDR MRSP clone, ST71, has a disrupted late genetic competence operon *comG* due to site-specific integration of a prophage, which we have named SpST71A. The disrupted *comG* likely serves as a novel genetic barrier to HGT in the ST71 S. pseudintermedius clone, which also perfectly correlates with its highly clonal population structure.

## RESULTS AND DISCUSSION

### WGS accurately predicts antibiotic resistance phenotype in U.S. isolates.

**FIG 1:**
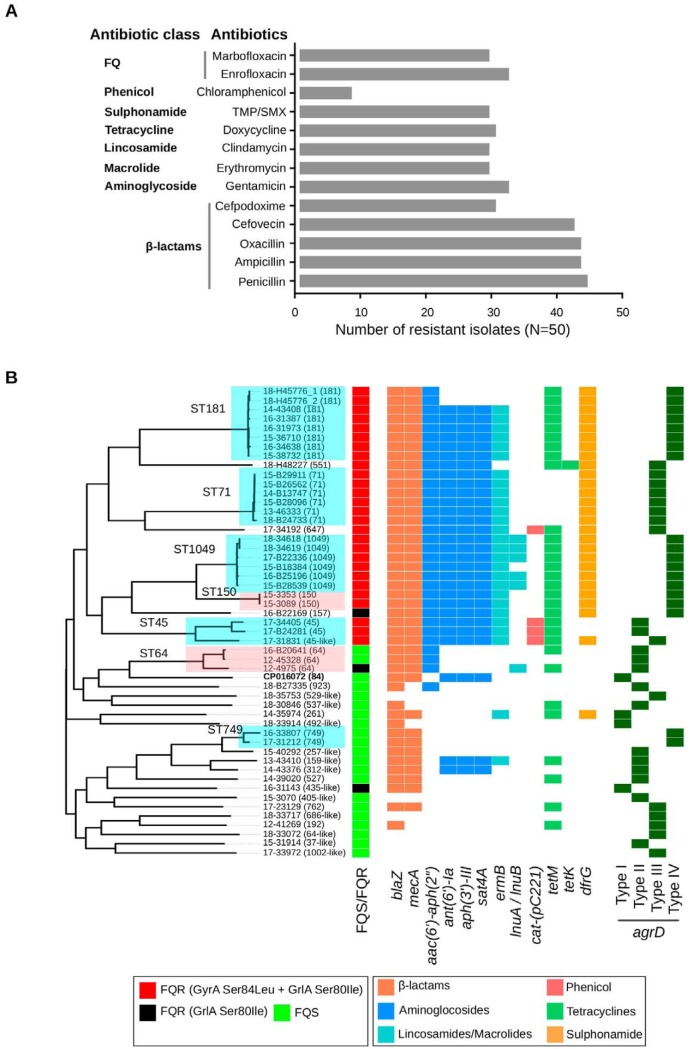
Antibiotic resistance profile and whole-genome ML phylogeny of US isolates. (A) Phenotypic susceptibility profile of 50 S. pseudintermedius isolates tested against multiple classes of antibiotics. (B) Whole-genome ML phylogeny of the 50 S. pseudintermedius isolates showing the presence and absence of acquired antibiotic resistance and all four types (type I to IV) of *agrD* genes. The presence (colored block) and absence (white space) of genes are depicted as a heat map, with color keys explained in the figure. The fluoroquinolone-resistant (FQR) isolates carrying GyrA Ser84Leu plus GrlA Ser80Ile mutations and only GrlA Ser80Ile mutation are shown in red and black, respectively. The FQ-sensitive (FQS) isolates without GyrA and GrlA mutations are shown in green (FQS/FQR column). The STs represented by two or more isolates in our data set are highlighted.

Phenotypic susceptibility testing of 50 clinical isolates against 22 antibiotics belonging to 7 different classes revealed a high prevalence of resistance in S. pseudintermedius. Resistance was found against 15 antibiotics, with 68% (34 of 50) of the isolates being multidrug resistant (Fig. 1A). As expected, resistance against β-lactam antibiotics was highest, followed by aminoglycosides, fluoroquinolones (FQs), tetracyclines, macrolides, lincosamides, and sulfonamides. While all 50 isolates were sensitive to rifampin and imipenem, chloramphenicol resistance was rare with only eight isolates being resistant to this drug (Fig. 1A). The resistance phenotype in most of the isolates perfectly correlated with the presence of a corresponding acquired resistance gene or mutation. For example, all isolates with phenotypic resistance to FQ carried GyrA Ser84Leu and GrlA Ser80Ile mutations (Fig. 1B). However, only four of the eight chloramphenicol-resistant isolates carried the *cat-pC221* gene. Consistent with previous studies, isolates with the *mecA* gene (henceforth, MRSP) were more likely to carry additional resistance genes and mutations, which results in multidrug resistance (8, 16). As shown, the most common STs in our data set were ST181, ST71 (European clone), ST1049, ST64, ST45, ST150, and ST749. None of the 50 isolates belonged to ST68, the most common epidemic clone in North America (Fig. 1B).

### Multidrug resistance in S. pseudintermedius correlates with sequence type.

**FIG 2:**
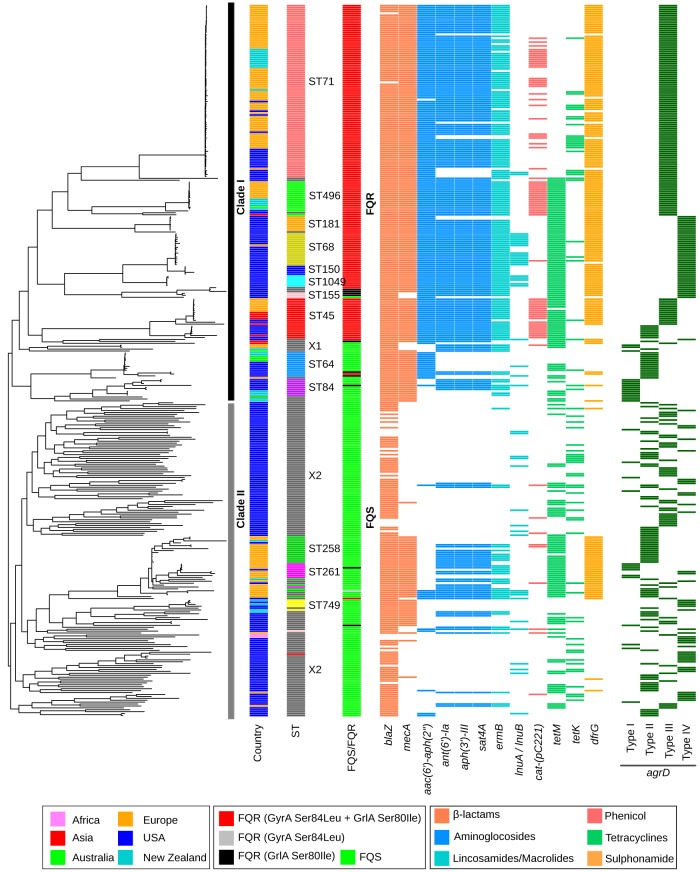
Whole-genome ML phylogeny of global S. pseudintermedius isolates. The 371 S. pseudintermedius genomes analyzed include 50 from this study and 321 publicly available published genomes. The color codes for the antibiotic resistance and *agrD* genes in this figure are the same as depicted in Fig. 1. The most frequently identified S. pseudintermedius clones from around the world are indicated (column ST). The country of origin for the isolates is also indicated by different colors (see key). Detailed information about the isolates and the identified resistance genes is provided in Tables S1 and S2 in the supplemental material, respectively.

Of the 371 genomes analyzed, 50 were sequenced as part of this study, whereas the remaining 321 were from publicly available genomes (see Table S1 in the supplemental material). The most common STs in the data set, in order of decreasing frequency, were ST71, ST45, ST496, ST68, ST258, ST64, ST84, ST181, ST1049, ST261, ST150, ST749, and ST155 (see Table S2). The maximum likelihood (ML) phylogeny, inferred based on the core genome alignment, assigned the isolates broadly into two major clades, clade I and clade II (Fig. 2). Clade I (*N* = 207) mainly comprised ST71, ST496, ST181, ST68, ST150, ST1049, ST155, ST45, ST64, and ST84 isolates, each forming distinct lineages within the larger clade. A few isolates in clade I were singleton STs that could not be grouped with any of the major STs. We called this heterogenous group “X1” for clarity. Clade II (*N* = 164), on the other hand, comprised ST258, ST261, and ST749 along with a large number (*N* = 128) of singleton STs, indicating that clade II is highly diverse with isolates from a wide range of genetic backgrounds (Fig. 2). This heterogenous group of isolates in clade II was called “X2.” The topology of the whole-genome phylogeny was consistent with the MLST grouping previously reported by Pires dos Santos et al. (10). Two recent studies have also obtained whole-genome phylogeny for S. pseudintermedius with a similar topology (8, 11).

10.1128/mSystems.00684-19.3 TABLE S1 Details of the 371 S. pseudintermedius genomes analyzed in this study. Download Table S1, XLSX file, 0.1 MB. Copyright © 2020 Brooks et al. 2020 Brooks et al. This content is distributed under the terms of the Creative Commons Attribution 4.0 International license.

10.1128/mSystems.00684-19.4 TABLE S2 Sequence type, antibiotic resistance genes, *agrD*, and CRISPR/Cas genes. Download Table S2, XLSX file, 0.1 MB. Copyright © 2020 Brooks et al. 2020 Brooks et al. This content is distributed under the terms of the Creative Commons Attribution 4.0 International license.

**FIG 3:**
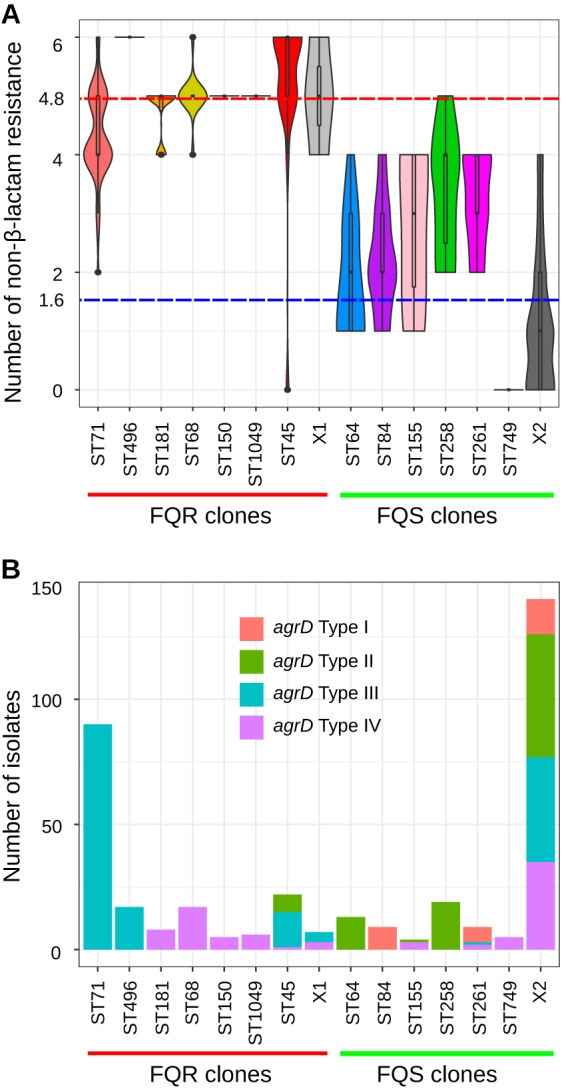
Analysis of antibiotic resistance and *agrD* genes. (A) Violin plot showing the distribution of non-β-lactam resistance genes. The red and blue dashed horizontal lines indicate the mean numbers of non-β-lactam resistance genes in clones belonging to FQR (mean = 4.8) and FQS (mean = 1.6), respectively. (B) Distribution of *agrD* type I to IV across different lineages of S. pseudintermedius.

The genomes were screened for the presence of acquired resistance genes using BLAST against the ARG-ANNOT (1,749 genes), ResFinder (3,077 genes), and NCBI (4,810 genes) databases (Table S2). No major differences were observed in the numbers and types of resistance genes predicted in the genomes when different databases were used. We also investigated these genomes for the occurrence of GyrA Ser84Leu and GrlA Ser80Ile, two well-characterized mutations conferring FQ resistance in bacteria. These mutations are widespread in FQ-resistant S. pseudintermedius isolates (14). It has been suggested that the emergence of FQ resistance has played an important role in the expansion of MRSP clones, similar to many methicillin-resistant S. aureus (MRSA) clones, including USA300 (24). Forty-seven percent (173 of 371) of the isolates in our data set, all belonging to clade I, contained GyrA plus GrlA mutations, henceforth called FQ resistant (FQR) (Fig. 2). This included MRSP clones ST71, ST496, ST181, ST68, ST150, ST1049, ST45, and the heterogenous group X1. The MRSP clones ST155, ST64, and ST84 in clade I did not carry GyrA/GrlA mutations and are henceforth called FQ sensitive (FQS). Similarly, all isolates in clade II, including MRSP clones ST258, ST261, and ST749, were FQS (Fig. 2). To better explain the results, we have divided the clones into FQR and FQS groups throughout the manuscript. All eight clones in the FQR group were MDR, containing four or more (mean = 4.8) non-β-lactam resistance genes, whereas only two clones in the FQS group, ST258 and ST261, were MDR (Fig. 3A). Many FQS isolates (the heterogeneous group X2) were *mecA* negative, henceforth, called methicillin-sensitive S. pseudintermedius (MSSP). These isolates were less likely to carry additional acquired resistance genes. The clones in the FQS group carried a significantly lower number (mean = 1.6) of non-β-lactam resistance genes than the clones in the FQR group (two-tailed *P* < 0.0001, Mann-Whitney test) (Fig. 3A). Overall, our analysis suggests that antibiotic resistance in S. pseudintermedius is largely correlated with sequence type (8, 10, 11, 21). Furthermore, the acquisition of FQ resistance appears to have played a key role in the evolution and clonal expansion of MRSP clones, similar to S. aureus (24).

### FQR clones harbor *agrD* type III or type IV.

The accessory gene regulator (*agr*) quorum sensing system plays an important role in regulating biofilm formation and virulence in staphylococci (20, 28). It is encoded by an operon consisting of *agrB*, *agrD*, *agrC*, and *agrA* genes (29). The *agrD* gene encodes a 45-amino-acid-long peptide, which after processing and maturation steps initiated by the AgrB protein, activates the membrane-bound histidine kinase AgrC. Activated AgrC phosphorylates AgrA, which in turn interacts with the cognate promoters to trigger *agr*-dependent transcription of the virulence-associated downstream genes, such as *hld* (δ-hemolysin) and *hla* (δ-hemolysin). Four types of *agrD* based on the autoinducing peptide (AIP) sequence variation have been identified in different S. pseudintermedius lineages (28). To determine if *agrD* type is correlated with STs and FQR, we investigated this gene in all 371 isolates. Since *agrD* is a core gene, all isolates exhibited 100% nucleotide sequence identity with one of the four *agrD* types described (28). However, our results showed that the clones in the FQR group predominantly contained type III or type IV *agrD* (Fig. 2 and 3B). In the FQR group, all ST71, ST496, and 66% of ST45 isolates carried type III *agrD*, while all ST181, ST68, ST150, and ST1049 isolates carried type IV *agrD*. While 33% of isolates in ST45 carried *agrD* type II, none of the isolates in the FQR group carried type I *agrD* (Fig. 3B). The isolates in the FQS group, on the other hand, mainly carried type I (ST84 and ST261) or type II (ST64 and ST258) *agrD*. Given the heterogenous structure of X2, this group included isolates with all four types of *agrD* (Fig. 3B; Table S2). These results are in agreement with a recent study showing a significant association between *agrD* type and MLST genetic type (20). Although no significant association was observed between *agrD* type and infection type, isolates with type II *agrD* were significantly more common in healthy dogs than in diseased dogs. Type II *agrD* isolates were also significantly less likely to be slime producers or to carry multidrug resistance and virulence genes than isolates with type I, III, or IV *agrD* (20).

### S. pseudintermedius possesses an open pangenome.

**FIG 4:**
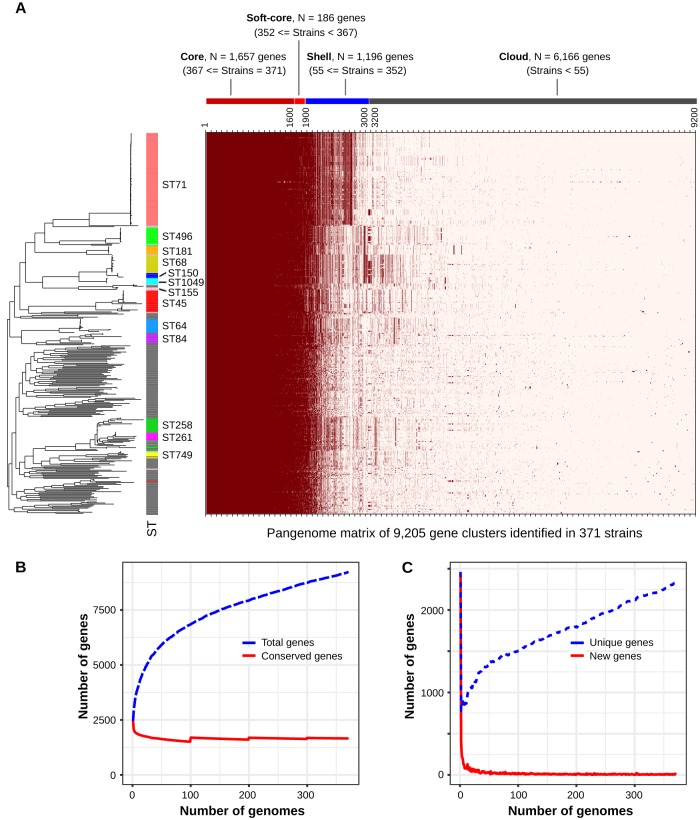
Pangenome analysis of S. pseudintermedius. (A) Pangenome matrix showing the presence and absence of gene clusters in all 371 genomes. The average number of genes per isolate was ∼2,515 (median = 2,502), of which ∼1,843 were core genes (combining both core [*N* = 1,657] and soft core [*N* = 186]). The pangenome size of S. pseudintermedius was estimated to be ∼9,205 (total number of genes identified combining all 371 isolates). The rows in the heat map correspond to isolates, and columns correspond to orthologous gene clusters. The dark red color means a gene is present in that isolate, whereas a light color denotes absence of the gene. The rows in the heat map are ordered according to the whole-genome ML phylogeny plotted with the heat map. (B) Rarefaction curves showing the estimated core and pangenome sizes of S. pseudintermedius by ROARY. The blue and red lines show the change in the number of total genes (pangenome size) and conserved genes (core genes), respectively, as new genomes are added in the analysis. (C) Rarefaction curves showing the trend of unique (blue) and new (red line) genes being discovered as new genomes are added in the analysis. As shown, the S. pseudintermedius pangenome is still open, as both pangenome size and the number of unique genes being discovered continue to increase after all 371 genomes have been added.

On average, each S. pseudintermedius isolate contained ∼2,492 protein-encoding genes (range, 2,252 to 2,820). The pangenome size (number of total gene clusters), based on the analysis of 371 genomes by ROARY, was estimated to be 9,205. Around 1,843 of them were classified as core genes (present in ≥95% of the isolates, combining core and soft core), 1,196 were classified as shell (present between 15% and 95% of the isolates), and the remaining 6,166 were classified as cloud (present in less than 15% of the isolates) (Fig. 4A). We also estimated the core and pangenome sizes of S. pseudintermedius using rarefaction and accumulation curve analyses (Fig. 4B). As depicted in the rarefaction curve, the pangenome size continuously increased with the addition of new genomes in the analysis, whereas the number of core genes becomes nearly constant at ∼1,850 after ∼100 genomes are analyzed (Fig. 4B). The number of unique genes also continuously increased with the addition of new genomes, with no indication of reaching a plateau (Fig. 4C). These results suggest that S. pseudintermedius, similar to many other *Staphylococcus* species, possesses an open pangenome (30–32). The pangenome matrix plotted with the whole-genome ML phylogeny indicated that most accessory genes (combining shell and cloud) were lineage specific (Fig. 4A). The presence of a large repertoire of accessory genes generally corresponds to the carriage of plasmids, prophages, and other mobile genetic elements (MGEs). The genes associated with specialized functions, such as antibiotic resistance, metal resistance, virulence, bacterial defense systems, and evasion of host immune systems, are most often part of the accessory gene repertoire (33). They also act as a reservoir for gene transfer to other bacterial species through the HGT mechanisms (31, 34). To gain insight into the potential function of the accessory genes identified in S. pseudintermedius, we performed a functional annotation analysis using eggNOG. As expected, a large number of these genes were related to prophages, conjugation system, CRISPR/Cas, restriction-modification (RM), antibiotic resistance, virulence, ABC-transporters, and DNA metabolism. We also found that the accessory gene content of FQR clones (mean = 758) was significantly higher (two-tailed *P* < 0.0001, Mann-Whitney test) than that of FQS clones (mean = 604) (see Fig. S1).

10.1128/mSystems.00684-19.1 FIG S1 Accessory gene content analysis. Violin plots showing the accessory gene content in S. pseudintermedius isolates. The dashed horizontal lines on the plot indicate the mean value in FQR (red, mean = 746), FQS (blue, mean = 603), and total (gray, mean = 676) isolates. Download FIG S1, EPS file, 0.4 MB. Copyright © 2020 Brooks et al. 2020 Brooks et al. This content is distributed under the terms of the Creative Commons Attribution 4.0 International license.

### Prophage content in S. pseudintermedius correlates with sequence type.

**FIG 5:**
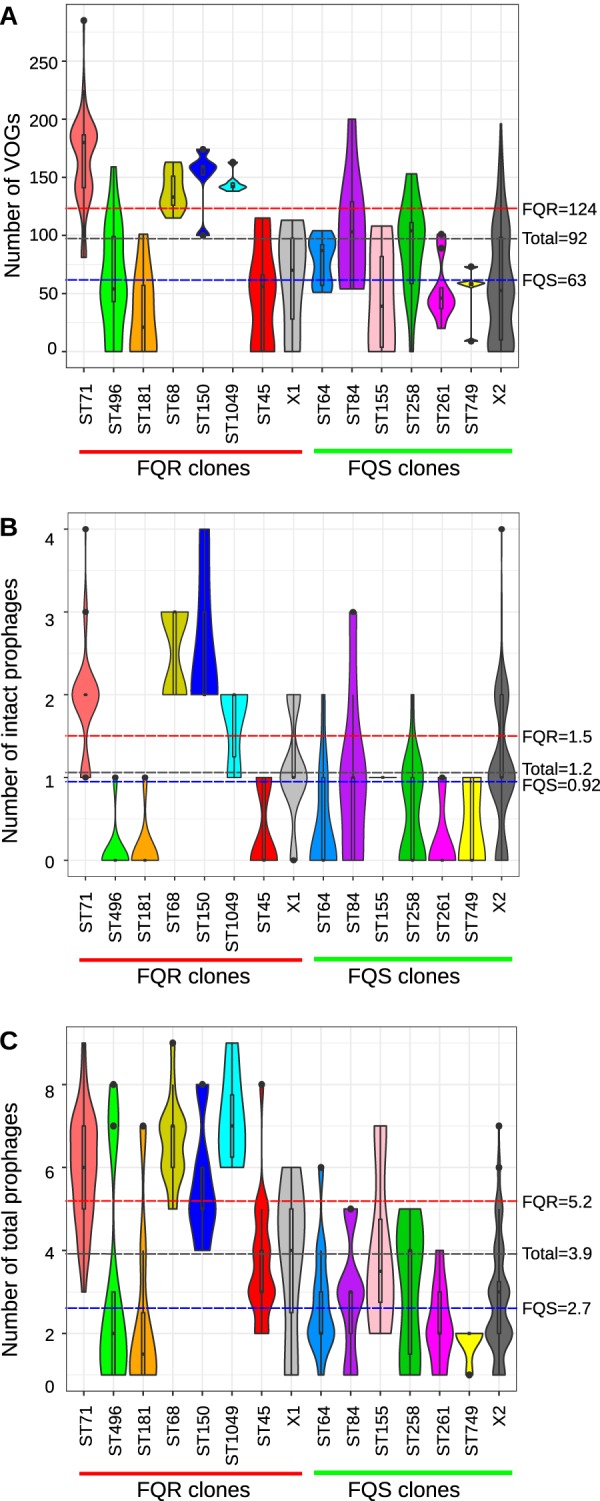
Prophage content analysis. Violin plots showing the number of prophages identified in S. pseudintermedius isolates. (A) Prokaryotic virus orthologous groups (pVOGs) identified using PHIGARO. (B) Intact prophages identified using PHASTER. (C) Total prophages (combining intact, incomplete, and questionable) across different lineages of S. pseudintermedius identified using PHASTER. The dashed horizontal lines on each plot indicate the mean values in FQR (red), FQS (blue), and total (gray) isolates.

Prophages are known to carry genes that contribute to bacterial fitness, virulence, resistance, and host adaptation (14). For example, the genes encoding the Panton-Valentine leucocidin (PVL) toxin and staphylococcal enterotoxin A (SeA) in the highly virulent S. aureus clone are harbored on an integrated prophage (35, 36). The chromosomal integration of ICE-emm12, carrying tetracycline and macrolide resistance genes, and prophage HU.vir, carrying SSA and SpeC superantigens in S. pyogenes, led to the selection and expansion of scarlet fever-associated clones in Hong Kong (23). Therefore, we investigated prophage sequences in all isolates, using two different methods. PHIGARO was used to quantitate the prophage content, while PHASTER was used to identify the intact prophage regions in each genome. The number of prophage-like genes, called pVOGs (prokaryotic virus orthologous groups) by PHIGARO, varied from 0 to 285 (mean = 92) per genome, indicating that some isolates did not harbor any prophages (Fig. 5A). The clones in the FQR group (mean = 124) had significantly higher pVOGs (two-tailed *P* < 0.0001, Mann-Whitney test) than FQS clones (mean = 63). Around 11% (42 of 371) of the isolates, mostly from the FQS group, did not show any pVOGs, suggesting that they did not carry any prophage (Table S3). PHASTER predicted intact prophages in ∼73% of the isolates, with an average of 1.2 intact prophages per genome (range 0 to 4). Like pVOGs, the number of intact prophages correlated with sequence type. ST71, ST68, ST150, and ST1049 carried significantly higher numbers of intact prophages than the rest of the FQR and FQS clones (two-tailed *P* < 0.0001, Mann-Whitney test) (Fig. 5B). No intact prophage was identified in ST496 and ST181. FQS isolates had on average 0.92 intact prophages per genome, which was significantly less than the average of 1.5 intact prophages in the FQR group (two-tailed *P* < 0.0001, Mann-Whitney test) (Fig. 5B). Most of the genomes in our data set were unfinished assemblies, and so there is a possibility that more isolates have intact prophages not identified by PHASTER. To address this issue, we analyzed questionable and incomplete prophages predicted by PHASTER (Fig. 5C). As shown, the number of total prophages was consistent with total pVOGs and intact prophage contents (two-tailed *P* < 0.0001, Mann-Whitney test) (Fig. 5A to C).

10.1128/mSystems.00684-19.5 TABLE S3 Details of pVOGs and prophages identified in each S. pseudintermedius. Download Table S3, XLSX file, 0.1 MB. Copyright © 2020 Brooks et al. 2020 Brooks et al. This content is distributed under the terms of the Creative Commons Attribution 4.0 International license.

### The epidemic clones ST71 and ST68 carry lineage-specific prophages.

**FIG 6:**
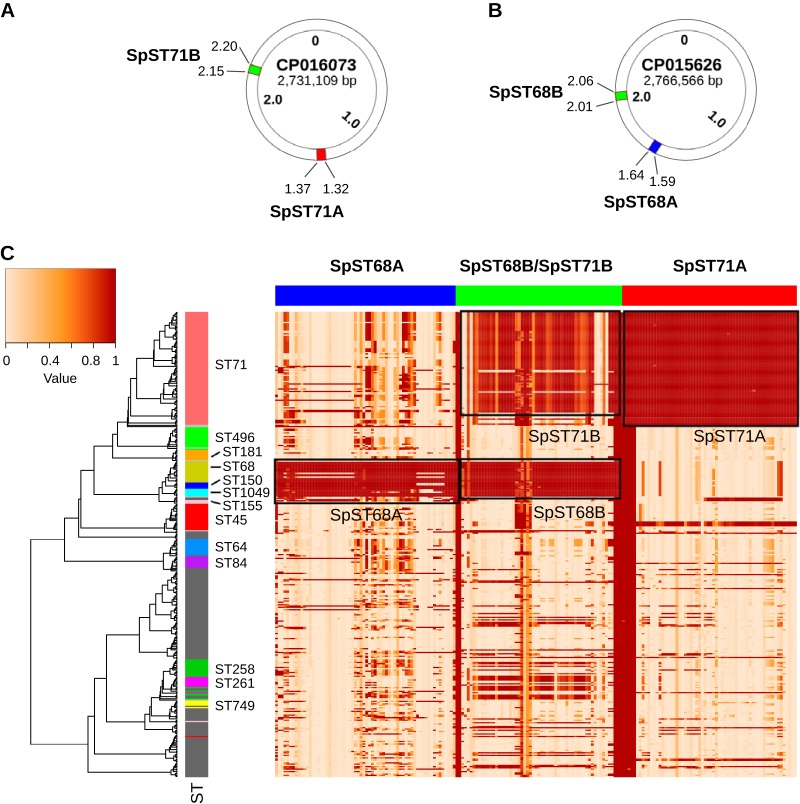
Distribution of ST71- and ST68-specific prophages. Circular maps showing the genomic coordinates (in Mb) of the intact prophages. (A) SpST71A and SpST71B in a representative ST71 genome. (B) SpST68A and SpST68B in a representative ST68 genome. (C) A heat map showing the distribution of four intact prophages across 371 genomes. The rows in the heat map correspond to isolates, and columns correspond to all the ORFs (genes) within the prophages. The color in the heat map is based on the BSR value (range 0 to 1) obtained by the LS-BSR analysis, with a darker color corresponding to prophage gene presence, and a lighter color corresponding to prophage gene absence. As shown, the SpST71A and SpST71B prophages are predominantly present in the ST71 lineage, whereas SpST68A and SpST68B are predominantly present in the ST68, ST150, ST155, and ST1049 lineages. The SpST71B and SpST68B are closely related prophages with ∼93% nucleotide sequence identity, which is also reflected in this gene presence/absence matrix.

We focused our analysis on the four intact prophages that were identified in ST71 and ST68 clones (Fig. 6A and B). They were named *Staphylococcus* phage SpST71A, *Staphylococcus* phage SpST71B, *Staphylococcus* phage SpST68A, and *Staphylococcus* phage SpST68B, following the bacteriophage naming guidelines of the Bacterial and Archaeal Viruses Subcommittee (BAVS) of the International Committee on the Taxonomy of Viruses (ICTV) (37). The unique identifiers SpST71A, SpST71B, SpST68A, and SpST68B reflect the STs in which they were predominantly present (Fig. 6A and B). The BLAST analysis using the large-scale blast score ratio (LS-BSR) revealed that SpST71A and SpST71B were present in all ST71 isolates, whereas SpST68A and SpST68B were present in ST68, ST150, ST1049, and ST155 isolates (Fig. 6C). It is important to note that ST68, ST1049, ST150, and ST155 are closely related STs, sharing the same lineage on the ML tree (Fig. 6C). As can be seen in the heat map, a few isolates belonging to other STs (such as 4 of 18 isolates in ST45) appear to have closely related regions homologous to these prophages (Fig. 6C). Furthermore, SpST71B and SpST68B were closely related prophages with ∼93% nucleotide sequence identity (Fig. 6C). In a recent study, Moodley et al. isolated four S. pseudintermedius prophages (vB_SpsS-SN8, vB_SpsS-SN10, vB_SpsS-SN11, and vB_SpsS-SN13) with almost identical morphology and high nucleotide sequence identity (38). These prophages did not show any significant homology with the four intact prophages identified in our study, suggesting that they were all different from each other (38). In another study, McCarthy et al. analyzed 15 S. pseudintermedius genomes (6 ST71, 2 ST260, and one each from ST68, ST261, ST263, ST262, ST309, ST25, and ST308) and reported three ST71-specific (ϕ1, ϕ2, and ϕ3) and four ST68-specific (ϕ3, ϕ6, ϕ7, and ϕ8) prophages (14). The genomic coordinates and functional annotation of these prophages, however, were not described in the paper (14). It is highly likely that the four prophages identified here are among the six prophages reported by McCarthy et al. (14).

### SpST71A is inserted within the competence operon *comG*.

**FIG 7:**
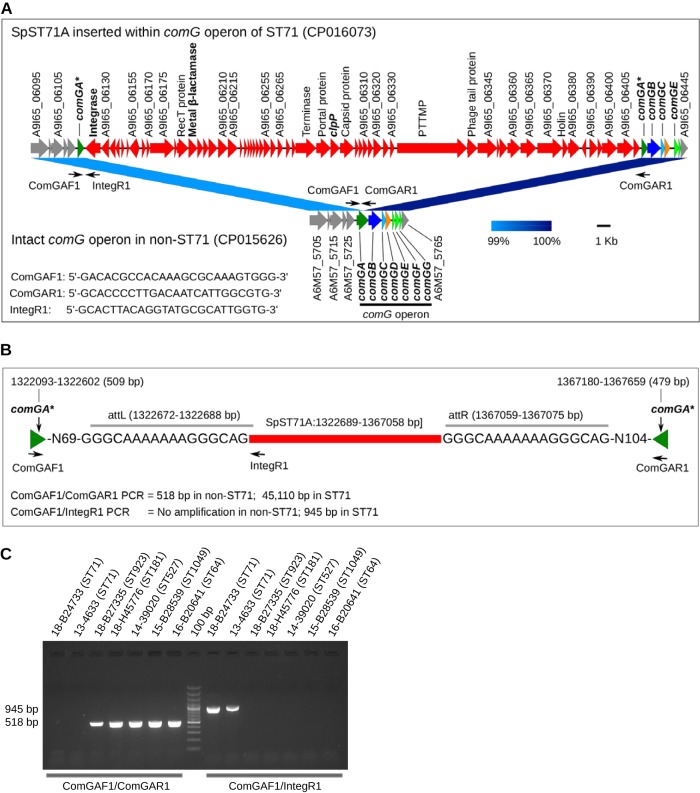
Genomic organization and PCR validation of SpST71A. (A) Chromosomal region of an ST71 genome (NCBI accession CP016073) with integrated SpST71A prophage shown in red. SpST71A is integrated in the middle of *comGA* (dark green arrow) splitting the gene into two parts, indicated by asterisks (*). The other ORFs of the *comG* operon (*comGB*, *comGC*, *comGD*, *comGE*, *comGF*, and *comGG*) are also indicated. An intact *comG* operon, without SpST71A prophage, in a non-ST71 genome (NCBI accession CP015626) is also shown for comparison. The ComGAF1, ComGAR1, and IntegR1 primers used for PCR screening of SpST71A are shown as small black arrows. The prophage ORFs whose functional annotations could not be predicted by eggNOG are identified by their locus tag. The functional annotation of all prophage ORFs is provided in Table S4 in the supplemental material. The linear comparison figure was created using Easyfig. (B) Schematic representation of the SpST71A insertion site and PCR amplification strategy. (C) A representative DNA agarose gel showing PCR screening of SpST71A prophage using ComGAF1/ComGAR1 and ComGAF1/IntegR1 primer pairs. Both set of PCRs were performed on all 50 isolates sequenced in this study. As shown, ComGAF1/ComGAR1 PCR could not amplify the 45,110-bp region (SpST71A) expected in ST71 but amplified the 518-bp *comGA* fragment in all non-ST71 isolates. ComGAF1/IntegR1 PCR amplified the 945-bp region in all ST71 isolates but not in other isolates. PTTMP, phage tail tape measure protein.

The process of natural DNA uptake in many bacteria relies on competence (Com) machinery, which is a complex system of proteins encoded by the late competence operons *comG*, *comE*, and *comF* (39). The functional expression of these operons is controlled by a master transcriptional activator gene, *comK*. Studies show that more than 80 species of bacteria carry fully functional Com machinery and therefore can take up exogenous DNA naturally (40). The most widely studied among them are Bacillus subtilis, Streptococcus pneumoniae, and Streptococcus mutans (39, 41–43). Studies have demonstrated that *com* genes, including *comG* and *comK*, are essential for DNA uptake, and functional inactivation of any of these genes renders bacteria naturally incompetent and nontransformable (41, 42). Our results showed that S. pseudintermedius has a complete *comG* operon, which consists of the *comGA*, *comGB*, *comGC*, *comGD*, *comGE*, *comGF*, and *comGG* genes (Fig. 7A). The *comGA* gene encodes an ATPase enzyme required for the assembly of pilin subunits and formation of the pseudopilus structure (39). A fully assembled pseudopilus facilitates the binding of exogenous DNA to the membrane-bound ComEA receptor, which in turn is transported across the cytoplasmic membrane though the ComEC channel with the help of an ATP-binding protein ComFA (39). For the first time, we have discovered that *comG* is disrupted in all S. pseudintermedius isolates belonging to ST71 (*N* = 90). The ∼44.3-kb SpST71A prophage is inserted within the *comGA* gene of the *comG* operon, splitting the 988-bp open reading frame (ORF) into two parts (5’-509 bp and 3’-479 bp) (Fig. 7A and B). A detailed analysis revealed that *comG* is also disrupted in 12 isolates belonging to other STs that included 4 of 18 ST45 and one isolate each from ST84, ST307, ST308, ST819, ST852, ST859, ST894, and ST901. This suggests that SpST71A, which is predominantly ST71 specific, may have spread to other lineages. Isolates belonging to all other STs in our data set had intact *comGA*. The *comK* gene was intact in all 371 S. pseudintermedius isolates irrespective of their genetic background. This is a significant finding, given the fact that natural genetic competence is one of the primary mechanisms of HGT in bacteria, along with conjugation and transduction. Further experimental studies are required to functionally validate the *comG* locus in S. pseudintermedius and to determine if prophage-mediated *comG* disruption contributes to the virulence and fitness of the ST71 lineage or aids in its clonal expansion. Prophage-mediated competence disruption is an extremely rare event in bacteria, with only one example reported in the literature to date (41, 44). Integration of a specific prophage A118 or ϕ10403S into *comK*, resulting in functional inactivation of this gene, has been identified in several Listeria monocytogenes strains (41, 44). As discussed previously, an intact and fully functional *comK* is necessary for the transcriptional activation of late competence genes. In a seminal study, Rabinovich et al. demonstrated that ComK and the other downstream competence proteins play a critical role in *Listeria* intracellular replication and virulence, in addition to their role in DNA uptake and competence (41). It has been shown that the prophage ϕ10403S is excised during phagosomal replication, leaving *comK* intact and functional, which activates the *Listeria* Com system. Activation of the Com system helps *Listeria* escape from the phagosome and infect neighboring cells. We hypothesize that the *comG* system may have a similar role in S. pseudintermedius replication.

10.1128/mSystems.00684-19.6 TABLE S4 Functional annotation of SpST71A, SpST71B, SpST68A, and SpST68B prophages. Download Table S4, XLSX file, 0.1 MB. Copyright © 2020 Brooks et al. 2020 Brooks et al. This content is distributed under the terms of the Creative Commons Attribution 4.0 International license.

Subsequently, we confirmed the *comGA* integration of the SpST71A prophage in 50 S. pseudintermedius isolates that were whole-genome sequenced in this study. Standard PCR with ComGAF1/ComGAR1 primers could not amplify the central 45,110-bp SpST71A sequence expected in ST71 (Fig. 7A to C). However, a 518-bp *comGA* sequence was amplified in all non-ST71 isolates, since their *comGA* gene was intact (Fig. 7C). The prophage-specific PCR with ComGAF1/IntegR1 primers, on the other hand, amplified the SpST71A-specific 945-bp band in ST71 but not in non-ST71 isolates. Thus, we have experimentally demonstrated that SpST71A prophage is inserted within the *comGA* gene (Fig. 7C). The ComGAF1/IntegR1 primers could be used for SpST71A prophage typing in future studies to investigate its presence in clinical MRSP isolates.

**FIG 8:**
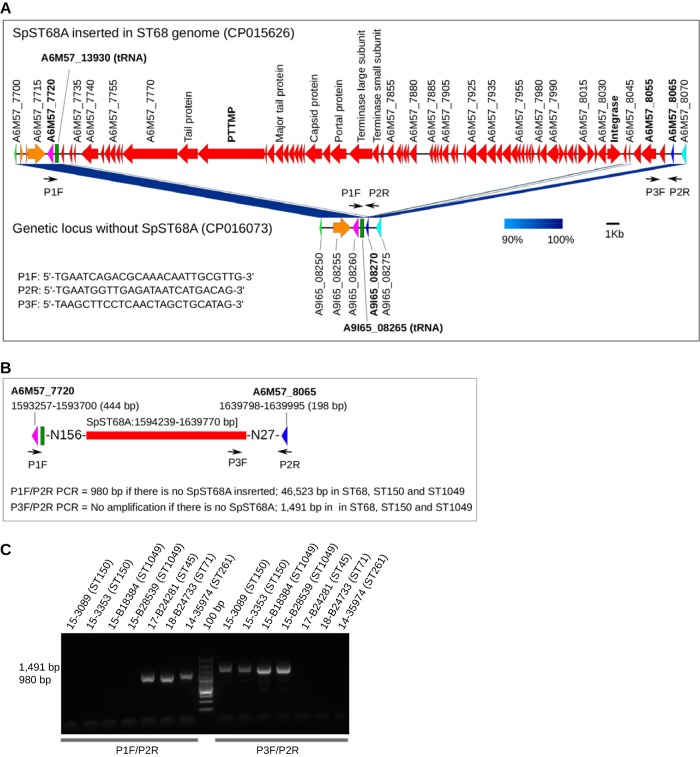
Genomic organization and PCR validation of SpST68A. (A) Chromosomal region of an ST68 genome (NCBI GenBank accession CP015626) with integrated SpST68A prophage shown in red. As indicated, the SpST68A is inserted between a tRNA locus (locus tag *A6M57_13930*) and a hypothetical gene (locus tag *A6M57_8065*). A non-ST68 genome (NCBI GenBank CP016073) without the SpST68A prophage is shown here for comparison. The P1F, P3F, and P2R primers used for PCR screening of SpST68A are shown as small black arrows. (B) Schematic diagram showing the SpST68A insertion site and PCR amplification strategy in a greater detail. (C) A representative DNA agarose gel showing PCR screening of SpST68A prophage using P1F/P2R and P3F/P2R primer pairs. Both set of PCRs were performed on all 50 S. pseudintermedius isolates sequenced in this study. As shown, P1F/P2R PCR could not amplify the 46,523-bp region (SpST68A) expected in ST150 and ST1049 but amplified a 980-bp region in isolates lacking SpST68A. P3F/P2R PCR amplified the 1,491-bp region in ST150 and ST1049 but not in other isolates.

The ∼45.5-kb SpST68A prophage was inserted between a tRNA locus (*A6M57_13930*) and a hypothetical gene (*A6M57_8065*) in an ST68 genome (Fig. 8A). After LS-BSR analysis, we found that SpST68A was also present in the ST150, ST1049, and ST155 lineages in addition to ST68 (Fig. 6C). Since none of the 50 isolates sequenced in this study belonged to ST68 or ST155, we could not confirm insertion of this prophage in ST68 and ST155 backgrounds. However, we were able to confirm SpST68A insertion in two ST150 and six ST1049 isolates that were available to us. The P1F/P2R primers could not amplify the 46,523-bp prophage sequence expected in ST150 and ST1049 but amplified a 980-bp central fragment in isolates lacking SpST68A (Fig. 8A to C). An SpST68A-specific PCR with P3F/P2R primers successfully amplified the 1,491-bp sequence in ST150 and ST1049 but not in other isolates (Fig. 8C). Like the SpST68A prophage, SpST68B and SpST71B were also found to be inserted between a tRNA locus and a hypothetical gene (Fig. S2).

10.1128/mSystems.00684-19.2 FIG S2 Genomic organization of SpST68B and SpST71B. The two nearly identical (∼93% nucleotide sequence identity) prophages SpST68B and SpST71B are inserted at the same genetic loci in their respective genomes. However, they have a few unique ORFs, both at the 5′ and 3′ ends, as indicated in this linear comparison figure. The functional annotation of all prophage ORFs is provided in Table S4. Download FIG S2, EPS file, 0.4 MB. Copyright © 2020 Brooks et al. 2020 Brooks et al. This content is distributed under the terms of the Creative Commons Attribution 4.0 International license.

### SpST71A, SpST71B, and SpST68B carry putative virulence and resistance genes.

Functional annotation of the intact prophages was performed using eggNOG to gain insight into their role in virulence and resistance (Table S4). The eggNOG results showed that the SpST71A prophage carried an ORF that encodes a putative class B metal β-lactamase (MBL) superfamily protein (A9I65_06190) and an ORF homologous to the *clpP* gene (A9I65_06290). MBLs are β-lactamase enzymes with a broad substrate spectrum that have been identified in many clinically important bacteria, including *Pseudomonas* and Acinetobacter (45). They can hydrolyze virtually all β-lactam antibiotics, except monobactams. The prophage-encoded *clpP* homologue is in addition to the core *clpP* gene (A9I65_10190) present in all 371 isolates irrespective of their sequence type (Fig. 7A). ClpP is a well-characterized protease in many organisms, including bacteria and parasites. In S. aureus and other bacteria, it has been shown to regulate many processes, including virulence, antibiotic resistance, biofilm formation, cell division, and stress response (46, 47). Interestingly, ClpP has also been found to inhibit genetic competence in S. mutans, B. subtilis, and L. monocytogenes. In S. mutans, MecA protein forms a complex with ClpC and ClpP to sequester and degrade SigX, a master regulator of genetic competence in this species (48). In B. subtilis, the master regulator ComK is sequestered and degraded by a ComK-MecA-ClpC/ClpP complex (49, 50). Thus, we show that the SpST71A prophage carries genes related to antibiotic resistance and virulence. The SpST68A prophage did not seem to carry homologues of any previously characterized virulence or resistance gene (Fig. 8A; Table S4). The two closely related prophages, SpST71B and SpST68B, however, carried a gene annotated as virulence-associated protein E (*virE*) (Fig. S2; Table S4). In addition to these three annotated virulence and resistance genes, there were many other hypothetical ORFs on the SpST71A, SpST68A, SpST71B, and SpST68B prophages that could not be annotated by eggNOG.

### Lineage-specific genetic barriers to HGT in S. pseudintermedius.

Bacteria have evolved various types of genetic barriers to HGT (51). They protect the host from invading foreign DNA, introduced by prophages and plasmids. The presence of these barriers, however, also makes genetic manipulation of the bacteria very difficult and sometimes impossible (52). The two most widely studied genetic barriers in bacteria are the restriction-modification (RM) and clustered regularly interspaced short palindromic repeats (CRISPR) associated with Cas protein (CRISPR/Cas) systems. As described above, we have discovered that *comG*, a genetic locus essential for natural genetic competence (natural DNA transformation) in many bacteria, is disrupted in all 90 ST71, 4 of 18 ST45, and eight singleton isolates belonging to minor STs, due to the integration of SpST71A prophage. This indicates that natural genetic competence, one of three major modes of HGT in bacteria, may not be functional in these lineages. Thus, the SpST71A-disrupted *comG* likely acts as an additional genetic barrier to HGT in S. pseudintermedius. The only other known example of the disrupted Com system in the literature is in L. monocytogenes, where the master transcriptional activator *comK* is interrupted by the insertion of a specific prophage A118 (41, 44). Next, we determined if the presence of RM and CRISPR/Cas systems in S. pseudintermedius were also lineage associated, as has been seen in other *Staphylococcus* species (34).

**FIG 9:**
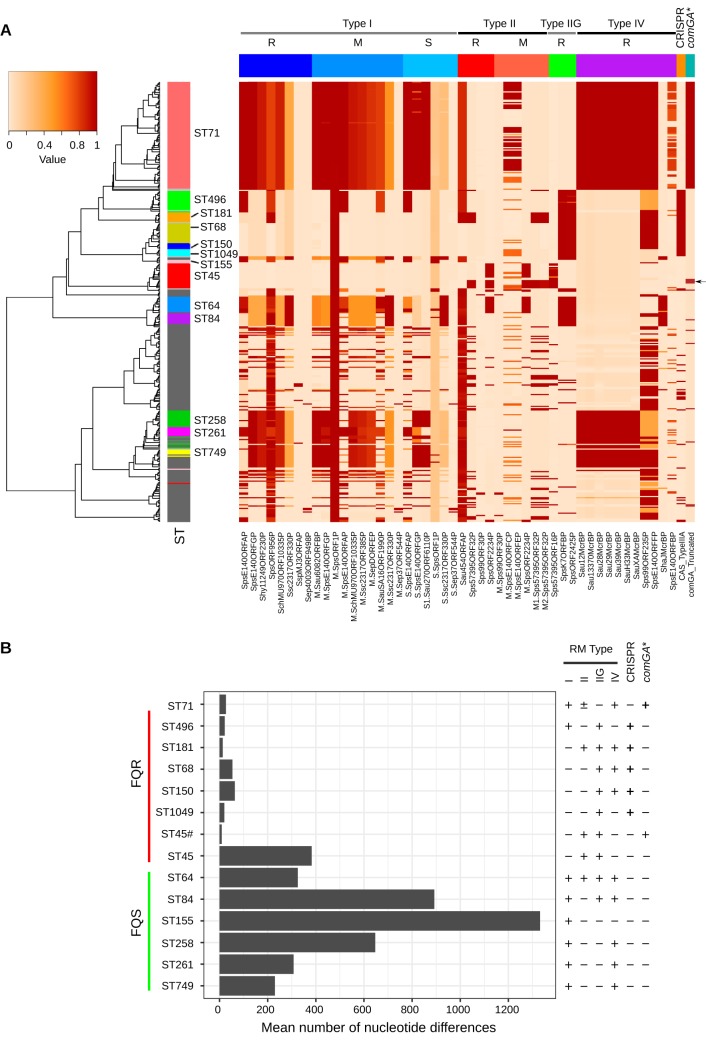
Genetic barriers to HGT in S. pseudintermedius. (A) A heat map showing the distribution of RM type I, type II, type IIG, type IV, CRISPR/Cas, and disrupted *comG* (comGA*) systems in 371 genomes. The rows in the heat map correspond to isolates, and columns correspond to genes. The restriction (R), modification (M), and specificity (S) subunits of RM types are indicated. The colors in the heat map are based on the BSR value (range 0 to 1) obtained by the LS-BSR analysis, with a darker color corresponding to gene presence, and a lighter color corresponding to gene absence. Four ST45 isolates with disrupted *comG* are indicated with the arrow. (B) Bar graph showing core genomic diversity (mean number of nucleotide difference across the core genome) within major lineages. The presence (+) and absence (−) of RM types, CRISPR/Cas, and disrupted *comG* are shown with each lineage. The clones containing CRISPR/Cas or disrupted *comG* exhibited extremely reduced genetic diversity compared to those lacking these systems. ST45#, four ST45 isolates with disrupted *comG*; ST45, remaining 14 ST45 isolates with intact *comG*. Please refer to Table S5 for recombination analysis of these clones.

Four major types of RM systems (types I, II, III, and IV) have been described in bacteria, based on their molecular composition, sequence recognition, and overall functionality (53). Type I RM systems comprise three enzymatic subunits: restriction endonuclease (R), DNA methyltransferase (M), and site specificity subunit (S) (54). Type II RM systems consist of R and M subunits, each with their own specificity functions (55). Unlike a typical type II RM, the type IIG RM system contains only one subunit, with all three functions combined. Type III RM systems contain R and M subunits, but the specificity function is only in the M subunit (56). Type IV RM systems contain only the R subunit and only cleave the modified DNA sequence (53). All four types of RM have been reported in *Staphylococcus* species (57). In S. aureus, RM systems, particularly type I, are major barriers for prophage and plasmid-mediated HGT (57, 58). Type I and type IV RM systems have also been identified as a major hindrance to genetic manipulation of staphylococci (53). Using REBASE, we have identified RM genes in all 371 isolates analyzed in this study, which is consistent with the fact that RM systems are ubiquitous in bacteria and archaea (Fig. 9A). Our analysis also indicated that RM types were lineage associated with most of the isolates containing more than one RM type (Fig. 9A and B). The ST68 clone predominantly contained type IIG and IV RM, while the ST71 clone contained type I and IV RM systems. However, 54 of the 90 ST71 isolates also contained type II RM system (Fig. 9B). As shown, type I RM was present in all clones in the FQS group. In contrast, only the ST71 and ST496 clones in the FQR group carried type I RM (Fig. 9B). Most importantly, type I and type IV RM systems were not identified in ST45 and ST1049 clones. Identification and characterization of RM systems in different MRSP clones will aid in developing strategies to genetically manipulate S. pseudintermedius.

10.1128/mSystems.00684-19.7 TABLE S5 Recombination parameters estimated using ClonalFrameML. Download Table S5, DOCX file, 0.1 MB. Copyright © 2020 Brooks et al. 2020 Brooks et al. This content is distributed under the terms of the Creative Commons Attribution 4.0 International license.

Unlike RM systems, the CRISPR/Cas system is not common in staphylococci; therefore, its role as a possible genetic barrier to HGT has not been studied closely in this genus. CRISPR/Cas system was detected in only 7% (29 of 430) of Staphylococcus epidermidis isolates analyzed in a study (59). Similarly, only 15% of coagulase-negative *Staphylococcus* (CoNS) species were found to carry CRISPR/Cas genes (60). The most common CRISPR/Cas identified in staphylococci is class 1 type IIIA, which contains *cas1-2*, *cas10*, *csm2-6*, and *cas6* genes (14, 34, 59). We detected CRISPR/Cas type IIIA in 24% of the isolates (87 of 371), all belonging to ST496, ST181, ST68, ST150, and ST1049. CRISPR/Cas was not detected in ST71 and ST45 isolates (Fig. 9A and B). CRISPR/Cas was also not found in any clone in the FQS group. Thus, barring 14 isolates in ST45, all other clones in the FQR group carried either CRISPR/Cas or disrupted *comG* as an additional genetic barrier to HGT (Fig. 9A and B; Table S5).

Furthermore, to determine if the presence of genetic barriers correlates with genome-wide nucleotide diversity and recombination, we estimated the average number of nucleotide differences per site (P) and parameters of recombination within each lineage (Fig. 9B; Table S5). Interestingly, the average nucleotide diversity perfectly correlated with the presence of CRISPR/Cas and disrupted *comG*. The average nucleotide diversity within isolates with CRISPR/Cas (all ST496, ST181, ST68, ST150, and ST1049 isolates) or disrupted *comG* (all ST71 and 4 of 18 ST45 isolates) was extremely low compared to that in the clones lacking these two systems (Fig. 9B). All lineages lacking these two systems exhibited high levels of nucleotide diversity. The relative contribution of recombination and mutation to the observed nucleotide diversity was estimated using ClonalFrameML (Table S5). The overall ratio of nucleotide substitutions introduced by recombination relative to mutation (r/m) in the core genome of S. pseudintermedius (*N* = 371) was estimated to be 1.74. Lineage-wise analysis of the r/m indicated that recombination has introduced 2 to 5 times more substitutions than mutation in most of the lineages (Table S5). The r/m value for ST71, ST68, and ST496, on the other hand, was less than one, suggesting that recombination has not contributed significantly to the nucleotide diversity of these lineages. The r/m value estimated in S. pseudintermedius is comparable to the values reported for S. aureus lineages, such as ST93 (r/m = 1.96), ST5 (r/m = 1.08), and ST239 (r/m = 1.13) (61–63). Furthermore, lineages with CRISPR/Cas or disrupted *comG* showed lower relative rates of recombination to mutation (R/θ) than the lineages without these barriers, thus suggesting the role of these systems in HGT and recombination.

The frequent carriage of prophages in S. pseudintermedius, especially in the FQR group, suggests that bacteriophage-mediated DNA transfer (transduction) is the major route of HGT in this species (64). As was mentioned previously, RM and CRISPR/Cas work as defense systems against bacteriophage infections. However, it is not clearly understood what mechanisms these prophages have utilized to overcome the host RM systems. Bacteriophages have evolved various strategies to evade bacterial RM systems (65). *Staphylococcus* prophage K, for example, uses restriction site avoidance to escape the host RM systems (65). Some prophages have acquired cognate methyltransferases to modify their own DNA sequence or antirestriction proteins to neutralize the host restriction endonuclease (65). Thus, the presence of RM systems does not make bacteria completely immune to bacteriophage infections. We do not know if prophages in S. pseudintermedius encode any such system to cross the host genetic barrier systems described above.

In conclusion, we show that (i) the prevalence of genes associated with antibiotic resistance, virulence, prophages, and genetic barriers to HGT differs significantly among S. pseudintermedius lineages; (ii) ST71 and ST68 clones carry lineage-specific prophages with novel virulence and antibiotic resistance genes; (iii) a key competence operon, *comG*, in the epidemic clone ST71 is disrupted due to insertion of the SpST71A prophage; and (iv) clones carrying CRISPR/Cas or SpST71A-disrupted *comG* show less nucleotide diversity and lower rates of recombination than clones lacking these two systems. Overall, our findings shed new light on the evolution and clonal expansion of MDR MRSP clones.

## MATERIALS AND METHODS

### Antibiotic susceptibility testing of 50 S. pseudintermedius clinical isolates.

The 50 S. pseudintermedius isolates sequenced in this study were obtained from clinical specimens submitted to the University of Illinois Veterinary Diagnostic Laboratory (VDL) between 2012 and 2018. All specimens were isolated from clinical infections. The specimens were grown overnight at 37°C on Columbia blood agar (CBA) (Remel Microbiology Labs, Thermo Fisher, Lenexa, KS), and the colonies were confirmed as S. pseudintermedius using traditional phenotypic tests such as colony morphology, Gram staining, and coagulase and catalase tests. Final identification of the species was performed using matrix-assisted laser desorption ionization–time of flight mass spectrometry (MALDI-TOF MS) analysis run in duplicates, and specimens with a confidence score of >1.8 were considered S. pseudintermedius. The phenotypic susceptibility (MIC) to 22 antibiotics (oxacillin, penicillin, amoxicillin, ampicillin, ticarcillin-clavulanic acid, cefoxitin, ticarcillin, cefazolin, cefpodoxime, ceftiofur, cefovecin, imipenem, enrofloxacin, marbofloxacin, gentamicin, amikacin, doxycycline, chloramphenicol, erythromycin, trimethoprim-sulfamethoxazole, clindamycin, and rifampin) was determined by broth microdilution (TREK Sensititre, Thermo Fisher, Lenexa, KS) and disk diffusion methods. The MIC results were interpreted according to the Clinical and Laboratory Standards Institute (CLSI) guidelines Vet-A04 and VetS-01 (CLSI 2013). An isolate was classified MRSP if it was phenotypically resistant to oxacillin (MIC ≥ 0.5 mg/liter) as recommended by the CLSI subcommittee on Veterinary Antimicrobial Susceptibility Testing (VAST). Isolates with oxacillin MIC of 0.5 mg/liter were confirmed resistant by phenotypic expression of PBP-2a using the MSRA detection kit (Denka Soikur Co. Ltd., Tokyo, Japan). Isolates with an intermediate level of susceptibility were considered resistant for the purpose of analysis, and those with resistance to three or more non-β-lactam antimicrobial classes were classified as MDR.

### Genomic DNA isolation, whole-genome sequencing, and assembly.

Genomic DNA was isolated from 1 ml of overnight culture, grown in tryptic soy broth (TSB) at 37°C with shaking at 200 rpm using the MasterPure Gram Positive DNA purification kit (Lucigen Corp., Middleton WI). Paired-end sequencing libraries were prepared with 1 to 2 μg of DNA by using the Nextera DNA Flex Library Preparation kit according to the standard Illumina chemistry and protocols. The libraries were quantitated by quantitative PCR (qPCR) and sequenced on one lane for 151 cycles from each end of the fragments (2 × 150-bp reads) on an Illumina HiSeq 4000 platform (Illumina Inc., San Diego, CA). The sequenced reads were assembled using the SHOVILL pipeline, which has TRIMMOMATIC for sequence reads cleaning and SPAdes v2.5.0 at its core for genome assembly (66, 67). In SHOVILL, the read depth reduction per sample parameter was set at 100× coverage of the estimated genome size. In addition to the 50 isolates sequenced in this study, we have also analyzed 321 publicly available published S. pseudintermedius genomes, mostly from the United States, Europe, Australia, and New Zealand (see Table S1 in the supplemental material). Twenty-one of 321 public genomes were downloaded as raw reads from the NCBI SRA database and assembled into contigs as described above. The remaining 300 genomes were downloaded as assemblies from the NCBI RefSeq database. The assembly quality (such as *N*_50_, number of total contigs, and genome size) of all 371 genomes was examined using the assembly-stats script (https://github.com/sanger-pathogens/assembly-stats). Assemblies with a total number of contigs of >150 or *N*_50_ of <40 kb were considered poor quality and were excluded from further analysis.

### MLST typing, genome annotation, and finding resistance and virulence genes.

The MLST of the isolates was determined from their genome assemblies using MLST-CHECK (https://github.com/sanger-pathogens/mlst_check), which utilizes blastn to compare the query sequences against all MLST profiles in the PubMLST (http://pubmlst.org/spseudintermedius/) database. The MLSTs were assigned clonal complexes (CC) using goeBURST, an optimized implementation of the eBURST algorithm (68, 69). The isolates sharing at least six identical alleles of seven were grouped into a single clonal complex (CC). Annotation of the genome assemblies was performed with PROKKA v1.5.2, excluding any contig less than 150 bp (70). All genome assemblies were screened for antibiotic resistance and virulence genes using ABRICATE (https://github.com/tseemann/abricate), which comes bundled with the ResFinder, ARG-ANNOT, NCBI, and CARD databases (71–73). A resistant or virulent gene was considered present in an isolate if it showed ≥80% sequence identity and ≥80% alignment coverage to the reference gene in the database. We also screened these genomes for the accessory gene regulator D (*agrD*), a widely studied and well-characterized gene associated with virulence in S. aureus and S. pseudintermedius (20, 28). The *agrD* homologues (NCBI GenBank accessions EU157356.1, EU157391.1, EU157400.1, and EU157402.1) were searched in genome assemblies using the LS-BSR (large-scale blast score ratio) with the TBLASTN option as described previously (25, 74).

### Pangenome analysis and whole-genome phylogeny.

The GFF3 files generated by PROKKA were used as input files for pangenome analysis using ROARY v.3.6.8, run with options -cd 99% (BLASTp percentage identity cutoff) -e and -mafft (75). Genes present in ≥95% of the genomes were classified as core genes, those present in ≥15% but <95% of the genomes were classified as shell genes, and the genes present in ≤15% of genomes were called cloud genes. The multi-FASTA core genome alignment produced by ROARY was subsequently used for phylogenetic analysis. Nucleotide positions predicted to be recombinant were identified using ClonalFrameML (76) and masked in the alignment using maskrc-svg *s*cript (https://github.com/kwongj/maskrc-svg). The putatively recombination-free alignment was used to infer the maximum likelihood (ML) phylogeny using RAxML-NG with the GTR gamma nucleotide substitution model and 200 bootstrap replicates (77).

### Identification of prophages in S. pseudintermedius genomes.

The putative prophage sequences in the S. pseudintermedius genomes were identified using PHIGARO (78). In PHIGARO, the supplied genome assemblies are first processed by Prodigal to call genes, which in turn are annotated with HMMSCAN using phage-specific profile hidden Markov models (HMMs) from pVOGs (prokaryotic virus orthologous groups), a comprehensive database of proteins from viruses that infect bacterial and archaeal hosts (79). A gene is considered “phage like” if it corresponds to one of the pVOG profile HMMs in the database. Prophage sequences within the genomes were also predicted using PHASTER (80). PHASTER classifies putative prophage regions as “intact,” “questionable,” or “incomplete” based on the proportion of phage genes in the identified prophage region. The four intact prophages identified in the ST71 and ST68 clones were screened in all other genomes using LS-BSR, as described in the previous section. The resulting bsr matrix was used to build a hierarchical clustering heat map using the hclust and heatmap.2 functions in R, with rows reordered according to the orders of taxa in the whole-genome ML phylogenetic tree. Functional annotations of the open reading frames (ORFs) in the predicted prophages (from PHASTER) and accessory genomes (from ROARY) were performed using the eggNOG database and the eggNOG-mapper tool (81, 82). The prophage comparison figures were created using Easyfig (83).

### PCR confirming lineage-specific integration of prophage SpST71A and SpST68A.

The integration of prophage SpST71A and SpST68A was first examined manually in the finished ST71 (NCBI GenBank accession CP016073) and ST68 (CP015626) genomes, respectively. Genome analysis indicated that SpST71A is integrated within the *comGA* gene, splitting the 988-bp ORF into two fragments. This integration was further confirmed using PCR with ComGAF1/ComGAR1 and ComGAF1/IntegR1 primer pairs. The ComGAF1 (5′-GACACGCCACAAAGCGCAAAGTGGG-3′) and ComGAR1 (5′-GCACCCCTTGACAATCATTGGCGTG-3′) primers were designed against the 5′ and 3′ ends of *comGA*, respectively, whereas the IntegR1 (5′-GCACTTACAGGTATGCGCATTGGTG-3′) primer was designed against the prophage integrase (*int*) gene. The integration of prophage SpST68A between *A6M57_13930* (tRNA locus) and *A6M57_8065* (hypothetical gene) loci of ST150 and ST1049 genomes was confirmed using PCR with P1F/P2R and P3F/P2R primer pairs. The P1F (5′-TGAATCAGACGCAAACAATTGCGTTG-3′) and P2R (5′-TGAATGGTTGAGATAATCATGACAG-3′) primers were designed against the *A6M57_13930* and *A6M57_8065* loci, respectively, whereas the P3F (5′-TAAGCTTCCTCAACTAGCTGCATAG-3′) primer was designed against the prophage locus *A6M57_8055*. All 50 clinical isolates sequenced in this study were screened for these two prophages. The cycling parameters for all four PCR amplifications were as follows: 95°C for 2 min, followed by 25 cycles of 95°C for 30 s, 55°C for 30 s, and 72°C for 1 min and a final extension of 72°C for 10 min.

### Identification of restriction-modification and CRISPR/Cas genes.

The genes homologous to RM systems were identified using the Restriction-ModificationFinder tool in combination with REBASE, a curated database of type I to IV restriction endonucleases, methyltransferases, and specificity units (84, 85). The genes encoding the CRISPR/Cas system were predicted using CRISPRCasFinder (86).

### Detection of genome-wide nucleotide diversity and recombination parameters.

The core genome alignment generated by ROARY, as described above, was used for estimating genome-wide average nucleotide diversity (mean number of nucleotide differences) across each lineage, using MEGA version 6 (87). ClonalFrameML was used to estimate the rates of recombination and mutation and their relative contribution to genetic diversity (76). The relative effect of recombination to mutation on the per-site substitution rate (r/m) was estimated using the formula (R/θ) × δ × ν. The R/θ ratio is the relative rate of recombination to mutation, δ is the mean length of DNA imported by homologous recombination, and ν is the divergence rate per site of DNA imported by homologous recombination (76).

### Data availability.

The raw sequence reads of the 50 isolates sequence in this study have been submitted to the NCBI’s Sequence Read Archive (SRA) database under the BioProject identifier (ID) PRJNA564152 and have also been supplied as supplementary information (Table S1).
