# Supplementary material for: Prophage-Mediated Disruption of Genetic Competence in Staphylococcus pseudintermedius
Source: mSystems. 2020 Feb 18;5(1):e00684-19. doi: 10.1128/mSystems.00684-19 (PMC7029219; doi:10.1128/mSystems.00684-19)
Supplement: TABLE S5 [file mSystems.00684-19-st005.docx]

**Table S5.** Recombination parameters estimated using ClonalFrameML

| **ST** | **FQR/FQS** | **CRISPR** | ***comGA**** | **N** | **R/θ (SD)** | **δ (SD)** | **ν (SD)** | **r/m** |
| --- | --- | --- | --- | --- | --- | --- | --- | --- |
| ST71 | FQR | − | + | 90 | 0.047 (6.37E-05) | 56.504 (8.95E-06) | 0.064 (6.50E-05) | 0.17 |
| ST496 | FQR | + | − | 15 | 0.074 (6.65E-04) | 166.037 (4.45E-06) | 0.054 (1.39E-04) | 0.62 |
| ST181 | FQR | + | − | 8 | 0.017 (2.78E-04) | 983.913 (1.02E-06) | 0.098 (9.57E-03) | 1.62 |
| ST68 | FQR | + | − | 15 | 0.052 (1.49E-04) | 101.833 (5.27E-06) | 0.053 (6.04E-05) | 0.28 |
| ST150 | FQR | + | − | 4 | 0.036 (1.31E-03) | 988.259 (1.01E-06) | 0.099 (9.69E-03) | 3.55 |
| ST1049 | FQR | + | − | 6 | 0.035 (5.80E-04) | 498.316 (1.96E-06) | 0.123 (3.76E-03) | 2.12 |
| ST45# | FQR | − | + | 4 | 0.034 (1.14E-03) | 987.469 (1.01E-06) | 0.009(9.67E-03) | 3.31 |
| ST45 | FQR | − | − | 14 | 0.849 (8.49E-01) | 499.249 (2.00E-03) | 0.009(9.45E-03) | 4.00 |
| ST64 | FQS | − | − | 13 | 0.753 (2.31E-03) | 584.922 (1.19E-08) | 0.007 (4.87E-08) | 3.06 |
| ST84 | FQS | − | − | 9 | 1.384 (3.28E-03) | 538.245 (5.91E-09) | 0.007 (2.20E-08) | 5.15 |
| ST155 | FQS | − | − | 3 | 0.762 (1.57E-03) | 404.009 (1.65E-08) | 0.010 (6.87E-08) | 3.14 |
| ST258 | FQS | − | − | 13 | 1.896 (3.53E-03) | 542.635 (3.33E-09) | 0.005 (9.77E-09) | 5.54 |
| ST261 | FQS | − | − | 6 | 1.137 (8.69E-03) | 516.302 (2.51E-08) | 0.007 (8.71E-08) | 3.90 |
| ST749 | FQS | − | − | 4 | 1.504 (1.71E-02) | 611.281 (2.03E-08) | 0.005 (6.04E-08) | 4.43 |
| Total |  |  |  | 371 | 0.815 (9.36E-06) | 246.605 (2.32E-10) | 0.009 (4.96E-10) | 1.74 |

*Note:* ST, sequence type; FQR, Fluoroquinolone resistant; FQS, Fluoroquinolone sensitive; N, number of isolates; R/θ, relative rate of recombination to mutation; δ, mean length of DNA imported by homologous recombination; ν, mean divergence rate per-site of DNA imported by homologous recombination; SD, standard deviations; The relative effect of recombination to mutation r/m = (R/θ) x δ x ν. ClonalFrameML calculates 1/δ, which was changed to δ. Total, all isolates analyzed in this study including minor STs. The presence (+) and absence (−) of CRISPR/Cas and disrupted *comG* (*comGA**) are shown with each lineage. ST45#, 4 ST45 isolates with disrupted *comG* (*comGA**); ST45, 14 ST45 isolates with intact *comG*.
